# Color-related chlorophyll and carotenoid concentrations of Chinese kale can be altered through CRISPR/Cas9 targeted editing of the carotenoid isomerase gene *BoaCRTISO*

**DOI:** 10.1038/s41438-020-00379-w

**Published:** 2020-10-01

**Authors:** Bo Sun, Min Jiang, Hao Zheng, Yue Jian, Wen-Li Huang, Qiao Yuan, Ai-Hong Zheng, Qing Chen, Yun-Ting Zhang, Yuan-Xiu Lin, Yan Wang, Xiao-Rong Wang, Qiao-Mei Wang, Fen Zhang, Hao-Ru Tang

**Affiliations:** 1 College of Horticulture, Sichuan Agricultural University, 611130 Chengdu, China; 2 Institute of Pomology and Olericulture, Sichuan Agricultural University, 611130 Chengdu, China; 3 Key Laboratory of Horticultural Plant Growth, Development and Quality Improvement, Ministry of Agriculture, Department of Horticulture, Zhejiang University, 310058 Hangzhou, China

**Keywords:** Molecular engineering in plants, Secondary metabolism, Transgenic plants

## Abstract

The carotenoid isomerase gene (*BoaCRTISO*) of Chinese kale was targeted and edited using the CRISPR/Cas9 system in the present study. The results showed a high mutation rate (81.25%), and 13 *crtiso* mutants were obtained. Only two types of mutations, insertions and replacements, were found. Both the total and individual carotenoid and chlorophyll concentrations of the biallelic and homozygous mutants were reduced, and the total levels declined by 11.89–36.33%. The color of the biallelic and homozygous mutants changed from green to yellow, likely reflecting a reduction in the color-masking effect of chlorophyll on carotenoids. The expression levels of most carotenoid and chlorophyll biosynthesis-related genes, including *CRTISO*, were notably lower in the mutants than in the WT plants. In addition, the functional differences between members of this gene family were discussed. In summary, these findings indicate that CRISPR/Cas9 is a promising technique for the quality improvement of Chinese kale and other *Brassica* vegetables.

## Introduction

Chinese kale (*Brassica oleracea* var. *alboglabra*) is a member of the Brassicaceae family, and the main edible parts of this plant are the highly nutritious tender leaves and bolting stems^1^. Our previous studies have shown that Chinese kale is rich in antioxidants and anticancer compounds, including vitamin C, glucosinolates, and carotenoids^2,3^. Carotenoids are a class of natural pigments that play important roles in photoprotection and antioxidant processes^4^. Their presence affects the color of many vegetables, fruits, and flowers, including those of tomato^5^, carrot^6^, cauliflower^7^, watermelon^8^, citrus^9^, and lily^10^.

Carotenoid isomerase (CRTISO), an enzyme that acts before the bifurcation point in the carotenoid biosynthetic pathway, is responsible for catalyzing the conversion of lycopene precursors to lycopene^11^. Previous studies have shown that the loss of function of *CRTISO* results in a yellow color in several crop species, including tomato^12^, rice^13^, and Chinese cabbage^14^. The rice *zebra* mutant accumulates lycopene precursors when growing in the dark and exhibits a “zebra” phenotype when growing in the light because the expression of the CRTISO gene *ZEBRA2* is reduced^13^. Su et al. found that the loss of function of the *BrCRTISO* gene caused an accumulation of lycopene precursors in and an orange phenotype of Chinese cabbage^14^.

Crop quality improvement is a topic of perennial importance in both food and agricultural research. However, traditional techniques for quality improvement, such as hybridization and chemical spraying, can be time consuming and inefficient. Because of its efficiency and convenience, the CRISPR/Cas9 system has been successfully used on many horticultural crop species to enhance plant resistance and improve yields. For instance, citrus resistance to canker was significantly improved by editing *CsLOB1* with the CRISPR/Cas9 system^15^. Targeted editing of *SlNPR1* was performed to alter the drought resistance of tomato^16^. Moreover, the heading stage of rice was accelerated, and the yield of oilseed rape was increased by editing *Hds*^17^ and *ALC*^18^, respectively. Recently, targeted editing of five tomato genes (*SGR1*, *LCY-B1*, *LCY-B2*, *LCY-E*, and *Bic*) were performed by Li et al.^5^ which blocked the biosynthesis of α- and β-carotene and increased the lycopene content by more than fivefold. Consequently, the nutritional quality of the edited tomato fruits improved. However, the application of CRISPR/Cas9 technology to the quality improvement of horticultural crops remains limited.

Recently, we established a CRISPR/Cas9 gene editing system in Chinese kale^19^ and used it to demonstrate functional differences among members of the *PDS* family, which are important genes involved in the carotenoid biosynthetic pathway^20^. Chinese kale is not typically colorful: the edible organs of most varieties are green, except for a few varieties that have red bolting stems^21^. Thus, the purpose of the present study was to perform targeted editing of *BoaCRTISO* using the CRISPR/Cas9 system to change the color and pigment concentrations of Chinese kale.

## Material and methods

### Plant materials and cultivation conditions

The typical white-flowered Sijicutiao cultivar of Chinese kale was used in this study. Sterile seedlings were grown on half-strength Murashige and Skoog (1/2 MS) media consisting of 0.8% Phytagar in a tissue culture chamber with 25/20 °C (day/night) temperatures, a 16/8 h (day/night) photoperiod, and a light intensity of 36 μmol m^−2^ s^−1^. After 7 d, cotyledons with 1–2 mm long petioles were cut and used as explants for *Agrobacterium*-mediated transformation.

### Plasmid construction

The primers used for plasmid construction are listed in Supplementary Table S1. The sequence of the *BoaCRTISO* gene (GenBank accession MN810158) was analyzed by using an online analysis tool (http://crispr.hzau.edu.cn/cgi-bin/CRISPR2/CRISPR), and a target site located on exon 11 with the sequence ACGTATGGACCAATGCCAAGAGG was selected; the underlined portion of the sequence is the protospacer adjacent motif of the target. We used a vector described in a previously published study^22^. CRTISO-CRISPR-F and CRTISO-CRISPR-R primers were obtained by removing AGG at the end of the target site, adding ATTG at the 5′ end of the target site, and adding AAAC at the 3′ end of the target site. The primers pairs CRTISO-CRISPR-F and CRTISO-CRISPR-R were then synthesized. To produce a vector that expressed both the Cas9 protein and guide RNAs, the primers pairs CRTISO-CRISPR-F and CRTISO-CRISPR-R were annealed, after which the primers and *Bsa*I-digested pAtU6-26-sgRNA-SK plasmids were mixed together to give rise to entry plasmids. The resulting recombinant plasmid rendered the sgRNA cassettes released by *Nhe*I and *Spe*I digestion. The cassettes were cloned into a pCAMBIA1300-*pYAO*:Cas9 binary vector previously digested by *Spe*I, followed by dephosphorylation. The resulting plasmids were used for transformation of wild-type (WT) plants (Supplementary Fig. S1).

### *Agrobacterium*-mediated transformation of Chinese kale plants

The transformation of Chinese kale was performed as described in our previous study^19^. Explants were precultured for 3 days in MS media consisting of 0.5 mg L^−1^ 2,4-D and 0.8% Phytagar, after which the explants were infected with the *Agrobacterium* strain GV3101 by immersion for 1–2 min. The explants were cocultivated with *Agrobacterium* for 3 d in MS media consisting of 0.03 mg L^−1^ naphthaleneacetic acid (NAA), 0.75 mg L^−1^ boric acid (BA), and 0.8% Phytagar. The explants were then transferred to MS media supplemented with 0.03 mg L^−1^ NAA, 0.75 mg L^−1^ BA, 0.8% Phytagar, 325 mg L^−1^ carbenicillin, and 325 mg L^−1^ timentin for 7 d. Hygromycin-resistant shoots that regenerated on the same media supplemented with 12 mg L^−1^ hygromycin B were transferred to tissue culture bottles that contained the subculture media. After 3 months, hygromycin-resistant plantlets were obtained, after which they were transplanted into trays containing a mixture of peat and vermiculite (3:1) within an artificial climate chamber with a light intensity of 80 μmol m^−2^ s^−1^, a temperature of 25/20 °C (day/night), a 12/12 h (day/night) photoperiod, and 75% relative humidity.

### Determination of transformation efficiency

Genomic DNA of both hygromycin-resistant and WT plants was extracted by the standard cetyltrimethylammonium bromide (CTAB) method^23^, and Hyg-F and Hyg-R specific primers (Supplementary Table S1) were designed based on the hygromycin gene sequence with the vector. Genomic DNA of each transgenic plant was used as a template for PCR-based amplification to screen the positive transgenic plants. Approximately 10 monoclones of each mutant were detected by sequencing. The PCR procedure was as follows: denaturation at 95 °C for 30 s, annealing at 56 °C for 30 s, and extension at 72 °C for 30 s. The transformants with the hygromycin gene were used for further analysis^19^.

### Detection of mutations

To evaluate mutations introduced into the CRISPR/Cas9 transgenic plants, the genomic DNA of each positive transgenic shoot was amplified using the specific primers CRTISO-CRISPR-test-F/R (Supplementary Table S1), which were designed to amplify the 730-bp flanking regions of the target site. The PCR products were then sequenced for genotypic analysis of the transgenic plants. The mutation rate was calculated, and all sequencing data were collected to analyze the mutation type^19^. Amino acid changes in the mutants were analyzed by DNAMAN 6.0 (Lynnon Biosoft, California, USA).

### Color measurements

The colors of the mutants were determined using an NR110 colorimeter (3nh, Shenzhen, China). The colorimeter was calibrated via a standard white plate according to the manufacturer’s instructions. Three positions on the leaves and the bolting stems of each mutant were randomly selected, and the L*, a*, and b* color values were obtained. The chromaticity (L*) represents the brightness (0–100:0 = black, 100 = white). Negative chromaticity (a*) indicates green, and positive chromaticity (a*) indicates red. Similarly, negative chromaticity (b*) stands for blue, and positive chromaticity (b*) stands for yellow. The a* and b* values are balanced toward 0, which represents white.

### Chlorophyll and carotenoid assays

Chlorophyll and carotenoid concentrations were determined using the methods of Shi et al.^24^. Two hundred milligrams of leaves or bolting stems was ground and extracted with 25 mL of acetone. The samples were sonicated for 20 min and then centrifuged at 4000 × *g* at room temperature for 5 min. The supernatant was filtered through 0.22 μm cellulose acetate filters and then analyzed by high-performance liquid chromatography (HPLC). HPLC analysis of the carotenoids and chlorophyll was carried out using an Agilent 1260 instrument equipped with a VWD detector. Samples (10 μL) were separated at 30 °C on a Waters C18 column (150 × 3.9 mm id; 3 μm particle size) using isopropanol and 80% acetonitrile-water at a flow rate of 0.5 mL min^−1^; the absorbance was measured at 448 and 428 nm.

### RNA extraction and qPCR expression analysis

The CTAB method^25^ was used to extract the total RNA from the leaves and bolting stems of mutant and WT plants. First-strand cDNA was synthesized by reverse transcription using a PrimeScript™ RT reagent kit with gDNA Eraser (Perfect Real Time, Takara). The qPCR primers used for the genes involved in the biosynthesis and degradation of carotenoids and chlorophyll in Chinese kale were designed based on *Brassica oleracea* primer sequences retrieved from the qPCR primer database (https://biodb.swu.edu.cn/qprimerdb/), except for the *CLH2*, *PPH*, and *NYC* genes^26^ (Supplementary Table S1). qPCR was performed following the procedures and recommended conditions of the TB Green Premix Ex Taq™ II qPCR Kit (Tli RNaseH Plus, Takara). Finally, fold changes in gene expression were calculated in terms of threshold cycles using the 2^−ΔΔCT^ method^27^, with the housekeeping gene *β-actin*^28^ serving as an internal reference. The expression level of *BoaCRTISO* in the WT bolting stems was set to one for genes that encode carotenoid biosynthesis enzymes. The expression levels of genes encoding carotenoid-degrading enzymes and chlorophyll biosynthesizing- and degrading-enzymes were calculated based on the respective expression levels of the respective genes in WT bolting stems.

### Statistical analysis

All the results are shown as the means ± standard deviations (SDs) of three replicates. Statistical analysis was performed using SPSS version 18 (SPSS Inc., Illinois, USA). The data were analyzed using one-way analysis of variance, and differences were compared using the least significant difference test at a significance level of 0.05.

## Results

### Analysis of *BoaCRTISO* mutations

Twenty-three hygromycin-resistant plants were obtained from ~2000 explants. No hygromycin target bands were observed in 7 out of the 23 plants (lines 1, 2, 5, 17, 18, 22, and 23) (Supplementary Fig. S2), indicating that the target expression cassette was transferred into the other 16 lines, and the transgenic efficiency was 69.57%.

**Fig. 1:**
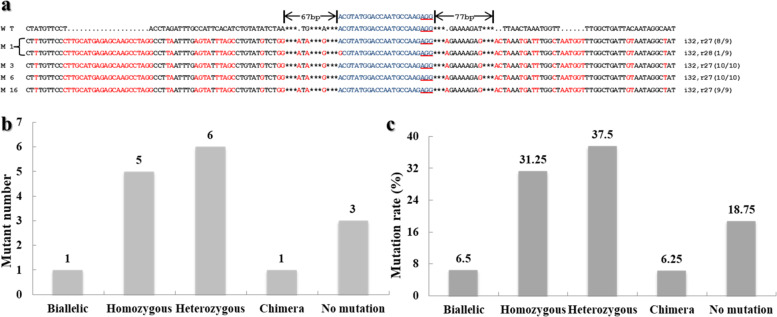
Vector map and *BoaCRTISO* mutations. **a** CRISPR/Cas9-induced mutations in Chinese kale. The target sequence is indicated in blue, the PAM sequence (NGG) is underlined in red, the mutated bases are indicated in red font, and the asterisks indicate spacing between bases. WT wild-type plant, M # number of mutants, i # number of base insertions, r # number of base replacements. M1 is a biallelic mutant with two kinds of sequences. M3, M6, and M16 are homozygous mutants with one kind of sequence. **b** Number of different mutant types. **c** Frequency of different mutant types

The sequencing results showed two mutation types, insertions and replacements, whereas no deletions were found. Combinatorial mutagenesis (32-bp insertions and 27-bp replacements occurring simultaneously) was predominant in these mutants, accounting for 97.40% of the total mutations, and other mutations (1- and 3-bp replacements) accounted for only 2.60% (Fig. 1a, Supplementary Fig. S3).

Thirteen out of the 16 transgenic plants harbored mutations, including one biallelic mutant that accounted for 6.25% of the total number of mutations among the transgenic plants, five homozygous mutants that accounted for 31.25%, six heterozygous mutants that accounted for 37.5%, and one chimeric mutant that accounted for 6.25%. Thus, biallelic and homozygous mutants accounted for 37.5% of the transgenic plants, and the percentage of transgenic plants with mutations was as high as 81.25% (Fig. 1). The base mutations caused several changes in the translation of the amino acids (Supplementary Fig. S4). The biallelic mutant M1 and the homozygous mutants M3, M6, and M16 were selected for subsequent studies.

### Color of *crtiso* mutants

**Fig. 2:**
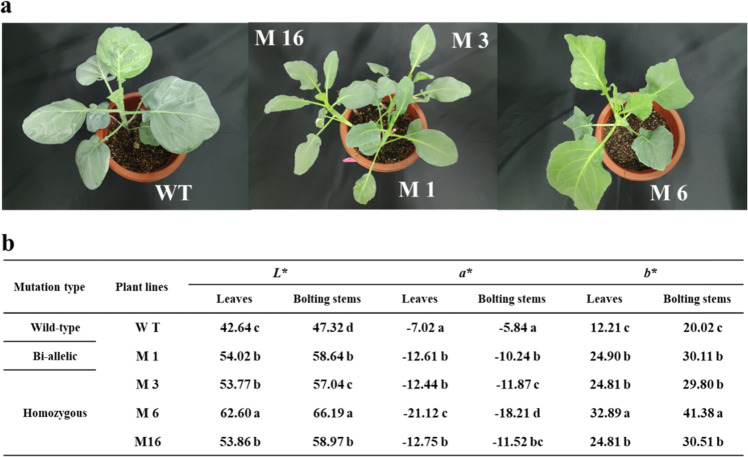
Phenotype of the *crtiso* mutants. **a** Phenotype of the *crtiso* mutants and wild-type plants at 6 weeks after transplanting. WT wild-type plant, M1 biallelic plant, M3, M6, M16 homozygous plants. **b** Color parameters of the *crtiso* mutants and wild-type plants at 6 weeks after transplanting. The data are expressed as the means of three replicates. The same letter in the same column indicates no significant differences among values (*p* < 0.05) according to the LSD test

Marked differences in color were observed between the *crtiso* mutants and WT plants. The leaves and bolting stems of the four tested mutants were yellow, with M6 being the most yellow (Fig. 2). The CILAB color parameters of the leaves and bolting stems of the WT and mutant plants were measured, the results of which are shown in Fig. 2b. The variation in chromatic parameters in the leaves and bolting stems of the four mutants was consistent. Specifically, the values of L* and b* were significantly higher than those of WT plants, while the values of a* were significantly lower. For example, the yellowness parameters (b*) of the leaves and bolting stems of M6 were 2.69- and 2.07-fold higher than those of WT plants (Fig. 2b).

### Pigment concentrations of *crtiso* mutants

**Fig. 3:**
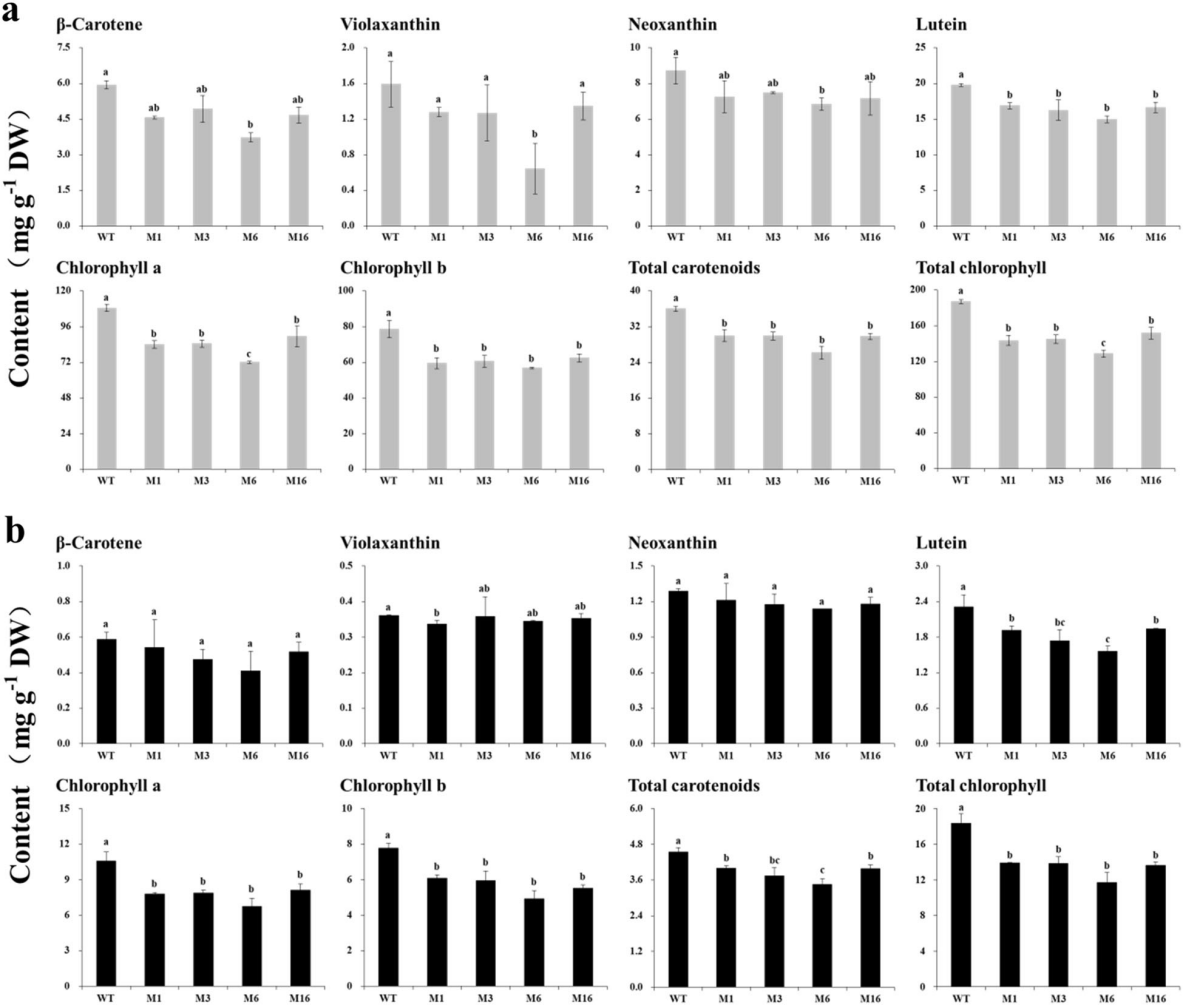
Pigment composition and concentrations in mutant and wild-type (WT) plants. **a** Concentrations of carotenoids and chlorophyll in the leaves of the mutants and WT plants at 6 weeks after transplanting. **b** Concentrations of carotenoids and chlorophyll in the bolting stems of the mutants and WT plants at 6 weeks after transplanting. WT wild-type plant, M1 biallelic mutant, M3, M6, M16 homozygous mutants. The data are expressed as the means ± SDs. The same letter in the same histogram indicates that there is no significant difference between the values tested, according to the LSD (*p* < 0.05)

Six individual pigments were measured: four carotenoids (β-carotene, violaxanthin, neoxanthin, and lutein) and two types of chlorophyll (chlorophyll a and chlorophyll b). The concentrations of individual and total carotenoids and chlorophyll in the leaves were significantly higher than those in the bolting stems. Chlorophyll a was the predominant pigment, while lutein was the major carotenoid in both the leaves and bolting stems (Fig. 3). The total carotenoid and chlorophyll concentrations in the mutants were significantly lower than those in the WT plants: the concentrations were more than 16% and 18% lower in the leaves, respectively, and more than 11% and 29% lower in the bolting stems, respectively. In particular, the M6 plant with the most obvious yellow phenotype had an ~25% lower total carotenoid concentration and a more than 30% lower total chlorophyll concentration in both the leaves and bolting stems.

The patterns of variation of individual pigments differed among the different organs. All pigments in the M6 leaves significantly decreased by more than 20% compared with those in the WT leaves. In particular, the violaxanthin concentration in the M6 leaves was 0.64 mg g^−1^ DW, 40% of that in the WT leaves. The concentrations of most pigments in the leaves of M1, M3, and M16 were also significantly lower than those in the WT leaves, although the differences in violaxanthin, β-carotene, and neoxanthin were not significant. The concentrations of lutein, chlorophyll a, and chlorophyll b in the bolting stems of the mutants were significantly lower than those of WT plants (by 16–32%, 23–36%, and 21–36%, respectively), whereas there were no significant differences in bolting stem concentrations of β-carotene, neoxanthin, or violaxanthin.

### Carotenoid- and chlorophyll-related gene expression levels in *crtiso* mutants

**Fig. 4:**
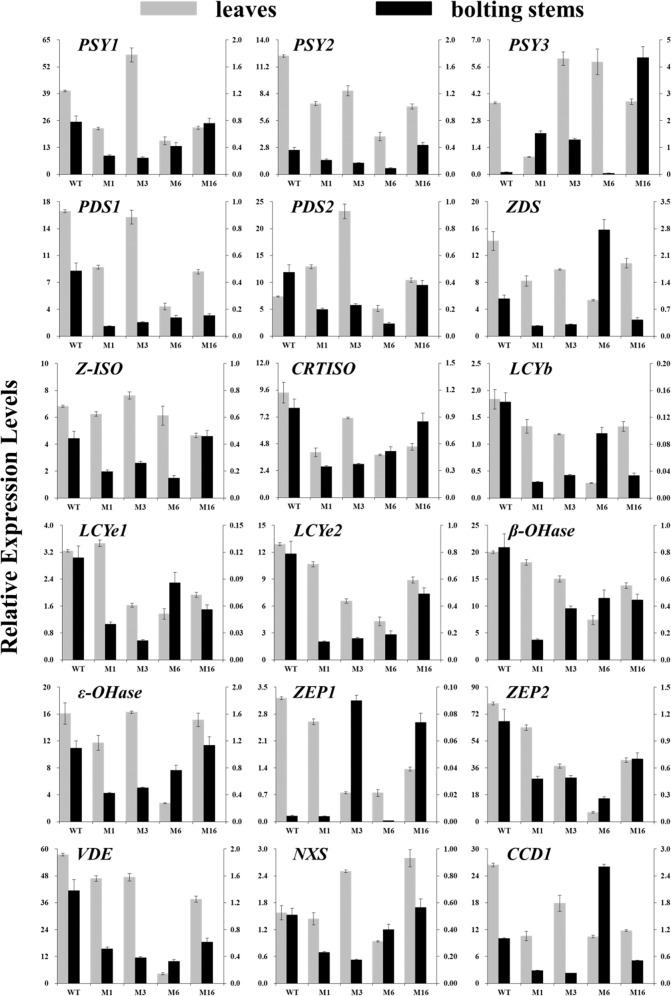
Expression levels of genes related to carotenoid biosynthesis and degradation. The leaves and bolting stems of mutants and WT plants were sampled 6 weeks after transplanting. The main axis represents the amount of gene expression in the leaves, and the secondary axis represents the amount of gene expression in the bolting stems. WT wild-type plant, M1 biallelic mutant, M3, M6, M16 homozygous mutants. GGPP geranylgeranyl diphosphate, PSY phytoene synthase, PDS phytoene desaturase, ZDS ζ-carotene desaturase, Z-ISO ζ-carotene isomerase, CRTISO carotenoid isomerase, LCYe lycopene ε-cyclase, LCYb lycopene β-cyclase, ε-OHase ε-carotene hydroxylase, β-OHase β-carotene hydroxylase, VDE violaxanthin de-epoxidase, ZEP zeaxanthin epoxidase, NXS neoxanthin synthase, CCD carotenoid cleavage dioxygenase

In WT plants, the expression levels of carotenoid biosynthesis-related genes in the leaves were substantially higher than those in bolting stems. The genes with the highest and lowest expression levels in the leaves were *ZEP1* and *NXS*, respectively, and they differed in their expression by ~50-fold. The genes with the highest and lowest expression levels in the bolting stems were *ZEP2* and *VDE*, respectively, and they differed in their expression by ~350-fold. Moreover, marked differences in gene expression were found among different members of the same gene family. The members with the highest expression levels in the *PSY*, *PDS*, and *LCYe* gene families were *PSY1*, *PDS1*, and *LCYe2*, respectively (Fig. 4).

After the targeted editing, the *CRTISO* gene expression in the leaves of the *crtiso* mutants was consistently downregulated. *CRTISO* expression was lowest in the M6 leaves, equal to 40.81% of that in the WT leaves. The *CRTISO* expression in the bolting stems of the mutants was also downregulated; M1 had the lowest *CRTISO* expression in the bolting stems—only 35% of that in the WT bolting stems (Fig. 4).

The expression levels of all carotenoid biosynthesis-related genes were measured, and the results indicated that the *BoaCRTISO* mutation led to reduced expression of most carotenoid biosynthesis-related genes in the leaves and bolting stems, although the degree of reduction varied widely (Fig. 4). Take M6 as an example. The expression of *ZEP*s in M6 leaves was downregulated by more than 90% compared with that in WT leaves, and the expression levels of most carotenoid biosynthesis-related genes (*PSY1*, *PSY2*, *PDS1*, *ZDS*, *CRTISO*, *LCYb*, *LCYe1*, *LCYe2*, *β-OHase*, *ε-OHase*, and *VDE*) were downregulated by 60–80%, whereas the expression levels of *PDS2* and *NXS* were downregulated by 30.62% and 40.51%, respectively. On the other hand, the expression of some genes whose products act upstream of the carotenoid biosynthetic pathway were upregulated rather than downregulated in the leaves and bolting stems of the mutants, although the upregulated genes were not identical in the leaves or bolting stems of a given mutant. Specifically, the expression levels of *PSY1* (M3), *PSY3* (M3 and M6), and *PDS2* (M1, M3, and M16) were clearly upregulated in the leaves, whereas the expression levels of *PSY3* (M1, M3, and M16) and *ZDS* (M6) were upregulated in the bolting stems. The expression level of *CCD1* in the *crtiso* mutant decreased significantly, except for the upregulation in M6 bolting stems. Nevertheless, the expression levels of *CCD4* were too low to be detected in both the WT and mutants.

**Fig. 5:**
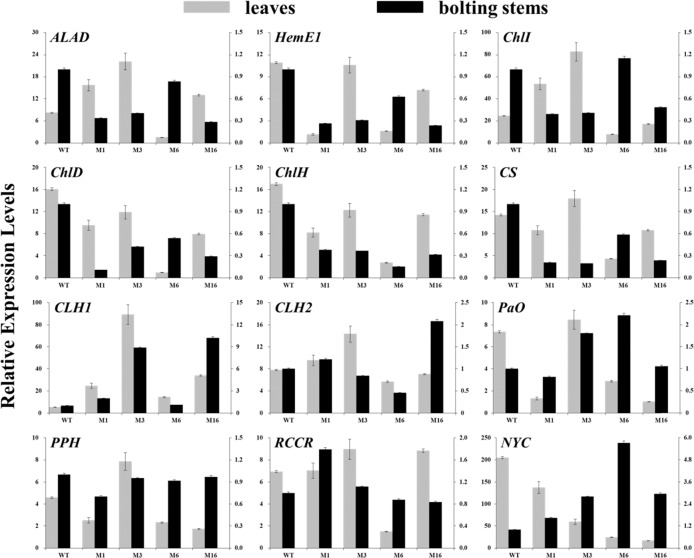
Expression levels of genes related to chlorophyll biosynthesis and degradation. The leaves and bolting stems of mutants and WT plants were sampled 6 weeks after transplanting. The main axis represents the amount of gene expression in the leaves, and the secondary axis represents the amount of gene expression in the bolting stems. WT wild-type plant, M1 biallelic mutant, M3 M6, M16 homozygous mutants. ALAD 5-aminolevulinic acid dehydratase, HemE1 glutamyl tRNA reductase, ChlI magnesium-chelatase I, ChlD magnesium-chelatase D, ChlH magnesium-chelatase H, CS chlorophyll synthase, CLH chlorophyllase, PaO pheide a oxygenase, PPH pheophytinase, RCCR red Chl catabolite reductase, NYC nonyellow coloring

The expression levels of chlorophyll biosynthesis-related genes in the leaves and bolting stems of *crtiso* mutants were generally downregulated (Fig. 5). The expression of chlorophyll biosynthesis-related genes in the leaves of M6 was reduced by more than 65%. However, some chlorophyll biosynthesis-related genes were upregulated. These genes included *ALAD* and *ChlI* in M1 leaves; *ALAD*, *ChlI*, and *CS* in M3 leaves; and *ChlI* in M6 bolting stems. The expression levels of genes encoding chlorophyll-degrading enzymes were also analyzed. The expression levels of *CLH1* in the leaves and bolting stems of all the mutants were upregulated, and the expression levels of *NYC* in the leaves and bolting stems were downregulated and upregulated, respectively. The expression levels of other genes encoding chlorophyll-degrading enzymes in the leaves and bolting stems varied from mutant to mutant (Fig. 5).

**Fig. 6:**
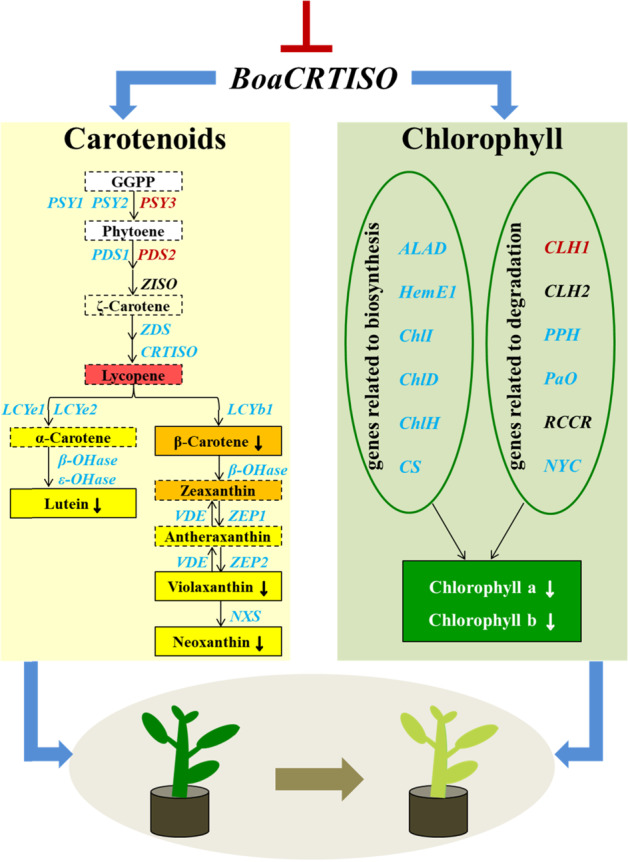
Schematic diagram of the results of this study. The solid frames indicate the presence of the substance in Chinese kale, and the dashed frames indicate the absence of the substance in Chinese kale. The blue genes were downregulated in the mutants, the red genes were upregulated in the mutants, and the black genes did not change significantly in terms of their expression. The down arrow next to a pigment indicates a decrease in its content. ⊥ indicates suppression

In summary, the results can be presented as a pattern, as shown in Fig. 6. The inhibition of *BoaCRTISO* gene expression in the *crtiso* mutants led to the downregulation of expression levels of carotenoid and chlorophyll biosynthesis-related genes, as well as carotenoid and chlorophyll contents, which led to the yellowing of the *crtiso* mutants.

## Discussion

In this study, the carotenoid isomerase gene from Chinese kale (*BoaCRTISO*) was targeted and edited using the CRISPR/Cas9 system and *Agrobacterium*-mediated stable transformation. As expected, 13 Chinese kale mutants were obtained, and the transgenic efficiency and mutation rates were 69.57% and 81.25%, respectively. The efficiency was relatively high compared with that in other plant species, such as tomato^16^, rice^18^, and watermelon^29^. The percentage of biallelic and homozygous mutants, the two most expected types of CRISPR/Cas9 mutants, reached 37.5%, which was substantially higher than that in previous reports^16,29^. Furthermore, *BoaCRTISO* was knocked down rather than knocked out in our study. Carotenoids are precursors of abscisic acid, which is essential for plant growth and stress resistance. Therefore, the knockdown of *BoaCRTISO* may be a better outcome than its knockout, as the latter may severely affect plant growth, development, and resistance to abiotic and biotic stress. These findings suggest that the CRISPR/Cas9 system is a promising and efficient technique for quality improvement in Chinese kale.

The concentrations of carotenoids and chlorophyll decreased in the *crtiso* mutants. The mutants also exhibited a yellow color, especially M6 (Fig. 2). In a previous study, the chlorophyll content of cucumber mutants decreased significantly, resulting in yellow leaves, which is consistent with our results^30^. We speculate that this is due to reduced chlorophyll concentrations and a concomitant reduction in their masking effect on carotenoid color. The color of a horticultural commodity is a primary characteristic perceived by consumers^31,32^. Vegetables with a higher chroma value and more vivid colors are typically judged to be fresher and of better quality than vegetables with dull colors^33^. The perception of color also affects other sensory perceptions, including taste^34^. Therefore, *crtiso* mutants of Chinese kale with a new color will likely have better market prospects in the future.

Carotenoids are crucial components of light-harvesting antenna complexes and play important roles in photosynthesis and photoprotection, especially the photoprotection of chlorophyll^35^. Our results showed that trends in chlorophyll reduction in the mutants were consistent with those of carotenoid reduction and were similar to those observed following virus-induced gene silencing of *LCYe* in tobacco plants^24^. This result may reflect photooxidative damage to chlorophyll caused by the reduction in the carotenoid content^35^. The chlorophyll content is influenced by the expression levels of genes related to chlorophyll biosynthesis and degradation^36^. In this study, the expression levels of chlorophyll biosynthesis-related genes significantly decreased, and the expression levels of several genes encoding chlorophyll-degrading enzymes were upregulated or unchanged in the *crtiso* mutants. In particular, the expression level of *CLH1*, a key gene that encodes a chlorophyll-degrading enzyme^26^, was notably induced in the mutants compared to the WT plants. All these changes resulted in a decrease in chlorophyll content.

CRTISO has emerged as a key regulatory step in the carotenoid biosynthetic pathway. When *CRTISO* expression is altered, the transcript level of other carotenoid genes can also be regulated^37^. In our study, the expression of most carotenoid biosynthesis-related genes in the *crtiso* mutants was downregulated. Similar results were found in the “zebra” mutant of rice: reduced *CRTISO* expression resulted in relatively low expression of all carotenoid genes except *VDE*^13^. *CCD1* and *CCD4* are the key genes that encode carotenoid-degrading enzymes in *Brassica*^38^. In the present study, the expression level of the *CCD1* gene was also significantly reduced, which may be a response to the decrease in the expression levels of carotenoid biosynthesis genes. In the *crtiso* mutants of Chinese kale, some genes differed significantly in their expression between the leaves and bolting stems. For example, the expression of *ZEP1* in the mutants was downregulated in the leaves but upregulated in the bolting stems. In the Chinese cabbage *crtiso* mutants, gene expression patterns in the inner, middle and outer leaves were not completely consistent^14^. These results suggest that the expression and regulatory patterns of genes differ among tissues.

The members of a gene family, having significant similarities in both structure and function, encode similar protein products. However, they are usually located at different positions on the same chromosome or on different chromosomes, each with different regulatory patterns and functions^39^. In our previous study, the two members of the *BoaPDS* gene family in Chinese kale (*BaPDS1* and *BaPDS2*) played different and indispensable roles, and their functions were partially complementary^20^. In this study, knockdown of *BoaCRTISO* resulted in downregulation of most carotenoid biosynthesis-related genes. However, some genes, such as *PSY3* and *PDS2*, had higher expression levels in the mutants. These results suggest that when the expression of one gene family member is inhibited, another member may be induced as a compensatory mechanism. However, the functional differences between these gene family members have not been determined and require further study.

In conclusion, this study used the CRISPR/Cas9 system to edit the *BoaCRTISO* gene of Chinese kale, resulting in the production of 13 mutants of the biallelic, homozygous, heterozygous, and chimeric types. Only insertion and replacement mutations were observed. The expression levels of carotenoid and chlorophyll biosynthesis-related genes in the *crtiso* mutants were significantly downregulated. Inhibition of *CRTISO* expression affected both the carotenoid and chlorophyll pathways, leading to decreased carotenoid and chlorophyll concentrations and creating a new yellow color of Chinese kale, with improved market prospects. The CRISPR/Cas9 system is therefore a promising technique for crop quality improvement.

## Supplementary information

Sun_et_al_revised Supplementary information

Clean version Sun et al. Supplementary information
