# Supplementary material for: Color-related chlorophyll and carotenoid concentrations of Chinese kale can be altered through CRISPR/Cas9 targeted editing of the carotenoid isomerase gene BoaCRTISO
Source: Hortic Res. 2020 Oct 1;7:161. doi: 10.1038/s41438-020-00379-w (PMC7527958; doi:10.1038/s41438-020-00379-w)
Supplement: Supplementary file 1 — Sun_et_al_revised Supplementary information [file 41438_2020_379_MOESM1_ESM.docx]

**Title page**

**Color-related chlorophyll and carotenoid concentrations of Chinese kale are altered through CRISPR/Cas9 targeted editing of the carotenoid isomerase gene *BoaCRTISO***

Bo Sun^1^, Min Jiang^1^, Hao Zheng^1^, Yue Jian^1^, Wen-Li Huang^1^, Qiao Yuan^1^, Ai-Hong Zheng^1^, Qing Chen^1^, Yun-Ting Zhang^2^, Yuan-Xiu Lin^2^, Yan Wang^2^, Xiao-Rong Wang^2^, Qiao-Mei Wang^3^, Fen Zhang^1, *^, Hao-Ru Tang ^1, 2, *^

^1^ *College of Horticulture, Sichuan Agricultural University, Chengdu 611130, China*

^2^ *Institute of Pomology and Olericulture, Sichuan Agricultural University, Chengdu 611130, China*

^3^ *Key Laboratory of Horticultural Plant Growth, Development and Quality improvement, Ministry of Agriculture, Department of Horticulture, Zhejiang University, Hangzhou 310058, China*

These authors contributed equally: Bo Sun, Min Jiang, Hao Zheng

Supplemental Information

Supplementary table 1: Primers used in the study

| Primer names | Sequence of primers (5’-3’) | Aims |
| --- | --- | --- |
| CRTISO-CRISPR-F | ATTGACGTATGGACCAATGCCAAG | Synthesis of the target site |
| CRTISO-CRISPR-R | AAACCTTGGCATTGGTCCATACGT |  |
| Hyg-F | CGATTGCGTCGCATCGACC | Detection of the hygromycin resistance gene |
| Hyg-R | TTCTACAACCGGTCGCGGAG |  |
| CRTISO-CRISPR-test-F | ACCTCTTCCATCGAAGATTGGGAG | Detection of the mutation in transgenic plants |
| CRTISO-CRISPR-test-R | ACCAAGAAGACCAGCATCAAGTACC |  |
| β-actin qRT-PCR F | CCAGAGGTCTTGTTCCAGCCATC | Detection of gene expression in mutants |
| β-actin qRT-PCR R | GTTCCACCACTGAGCACAATGTTAC |  |
| PSY1-qPCR-F | AAGGGCTGTAGAGTCTTCTAGA |  |
| PSY1-qPCR-R | CGTTTTTGTTGTTTGCTTCCTC |  |
| PSY2-qPCR-F | GGGACCTTGATGATCTGAAGAA |  |
| PSY2-qPCR-R | TTCACCCAACAAACTCAAACTC |  |
| PSY3-qPCR-F | TTCATGAGACTGCAGCTTAAGA |  |
| PSY3-qPCR-R | CCCAACATAAGCTCTCTTGGTA |  |
| PDS1-qPCR-F | GATCTCTTCACAAGCGCTTAAG |  |
| PDS1-qPCR-R | GCTTCCAAGAAATTGACAGTGT |  |
| PDS2-qPCR-F | TCATCTGGAGGTTGTGATTTGA |  |
| PDS2-qPCR-R | ATATCCACACAAACTACCTGCA |  |
| ZDS-qPCR-F | CGTTCCCTTGTCCCGAGCAA |  |
| ZDS-qPCR-R | CCTCGGAGGTTTCATGTTAGGTCTTC |  |
| ZISO-qPCR-F | CGTTGTTTGGATCGATAACTCC |  |
| ZISO-qPCR-R | GCTAATCCACTATGCACAGTTG |  |
| CRTISO-qPCR-F | ATATCCACACAAACTACCTGCA |  |
| CRTISO-qPCR-R | AGATTGGGAGGGACTCACTCCA |  |
| LCYb-qPCR-F | GTTGTTGATCTAGCTATCGTTGGC |  |
| LCYb-qPCR-R | GAGTTTGGGGGAAGGATCGAT |  |
| LCYe1-qPCR-F | GGTTTGTGTAGTAGAGTCGTCA |  |
| LCYe1-qPCR-R | TCAACGAGCTTAGACTGTTCAT |  |
| LCYe2-qPCR-F | CAGGTTCCGGTATAGAGAGTTG |  |
| LCYe2-qPCR-R | ACGTATAGAATCTCCGAACCAC |  |
| β-OHase-qPCR-F | CCTAATGGAGTGAAAAGCATCG |  |
| β-OHase-qPCR-R | TCTTTACTGTTGATGGGAAGCT |  |
| ε-OHase-qPCR-F | TCTCCTAAACCCAGATTCGTCTCC |  |
| ε-OHase-qPCR-R | GCGAGTGAGTGATGTGAGCCA |  |
| VDE-qPCR-F | AAGGCATTTCCTCACATCCTTA |  |
| VDE-qPCR-R | ACCAAGAAAGTGCCTTTGATTC |  |
| ZEP1-qPCR-F | AGAAGTCCTAGTTTCACTTGGG |  |
| ZEP1-qPCR-R | TCTGTTTCCATGCTTGTTCAAG |  |
| ZEP2-qPCR-F | AGAACTCCTAGTTTCACTTGGG |  |
| ZEP2-qPCR-R | TATCCTCAATGGCCATACATCC |  |
| NXS-qPCR-F | CAGTTGACATGCCAGCAAGTCC |  |
| NXS-qPCR-R | CGAATCGGATGATACACTGGGGA |  |
| ALAD-qPCR-F | AGCTTCCCATCGATCAAAGTAA |  |
| ALAD-qPCR-R | GAAGTTGCAACTGGAAGAGAAG |  |
| HemE1-qPCR-F | ATGTTACTCCTCAGGCTTATCG |  |
| HemE1-qPCR-R | AGAGTTAGTGTGTCTTCTTGGG |  |
| ChlI-qPCR-F | TCTCTTCTTCTTCCTCAACACC |  |
| ChlI-qPCR-R | CCTTATTTGGATTCCTGCGTTT |  |
| ChlD-qPCR-F | TCTCAACATATCGTCTCTTCCG |  |
| ChlD-qPCR-R | GGTGATTCGAAGATAGCATTCG |  |
| ChlH-qPCR-F | CACTACCACCAAACACTCATTC |  |
| ChlH-qPCR-R | GAGACAGAGGACTTCACTTTGA |  |
| CS-qPCR-F | GGCGGAGACTGATACAGATAAA |  |
| CS-qPCR-R | TACCAAGAAGCTGGTTAATGCT |  |
| CLH1-qPCR-F | TGTAATAATCACCTCCCCAACC |  |
| CLH1-qPCR-R | ACAATGTAGCCATGAGAAGCTA |  |
| PaO-qPCR-F | TCTCGATTTCTCCTCAAAGACC |  |
| PaO-qPCR-R | TGAACTCAGACCCTTCTTCTTC |  |
| RCCR-qPCR-F | GTACGGAACTTCAAGAACCCTA |  |
| RCCR-qPCR-R | ACTTTAAAACCAGCATGCAGAG |  |
| CHL2-qPCR-F | AGATGCCTGTTCTAGTTATTGG |  |
| CHL2-qPCR-R | CACGCTGGACCTTGACATTC |  |
| PPH-qPCR-F | AGAGGTTATCGGTGAGCCA |  |
| PPH-qPCR-R | GACGAGATGAGGATGGG |  |
| NYC-qPCR-F | TTACATCTCGCAGTTCTGA |  |
| NYC-qPCR-R | GCAATACCAACTACCTTAGC |  |
| CCD1-qPCR-F | CTCAAAGCTTCTCGATCTTGTG |  |
| CCD1-qPCR-R | CAACCCTCACAAATTCACCATT |  |
| CCD4-qPCR-F | TACACTCGAACCAACCATACCG |  |
| CCD4-qPCR-R | AAACACGTCCTCTCAGACAACT |  |


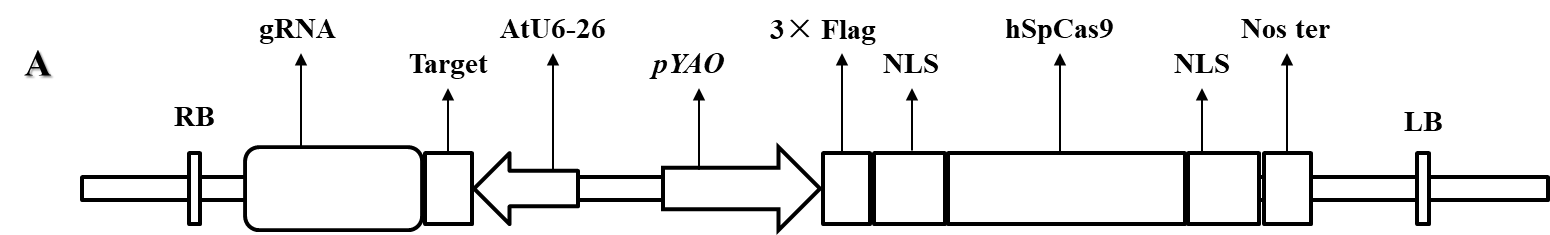


**Supplementary Fig. S1** Structure of the CRISPR/Cas9 binary vectors for Chinese kale transformation. The Cas9 cassette was driven by the *pYAO* promoter, while sgRNA was controlled by the AtU6 promoter. The 3 × Flag is a label which can be used to identify the transgenic plants. NLS, nuclear localization sequence.


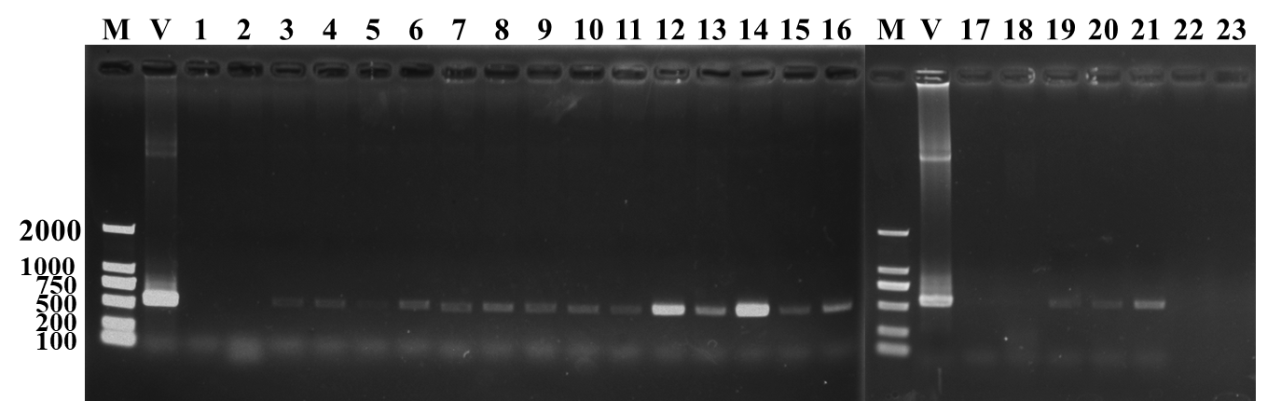


**Supplementary Fig. S2** PCR detection of the hygromycin-resistant gene for the estimation of transformation efficiency. M: DL2000 maker; V: empty vector was used as a positive control; 1–23: indicates the resistant plant line number.

**
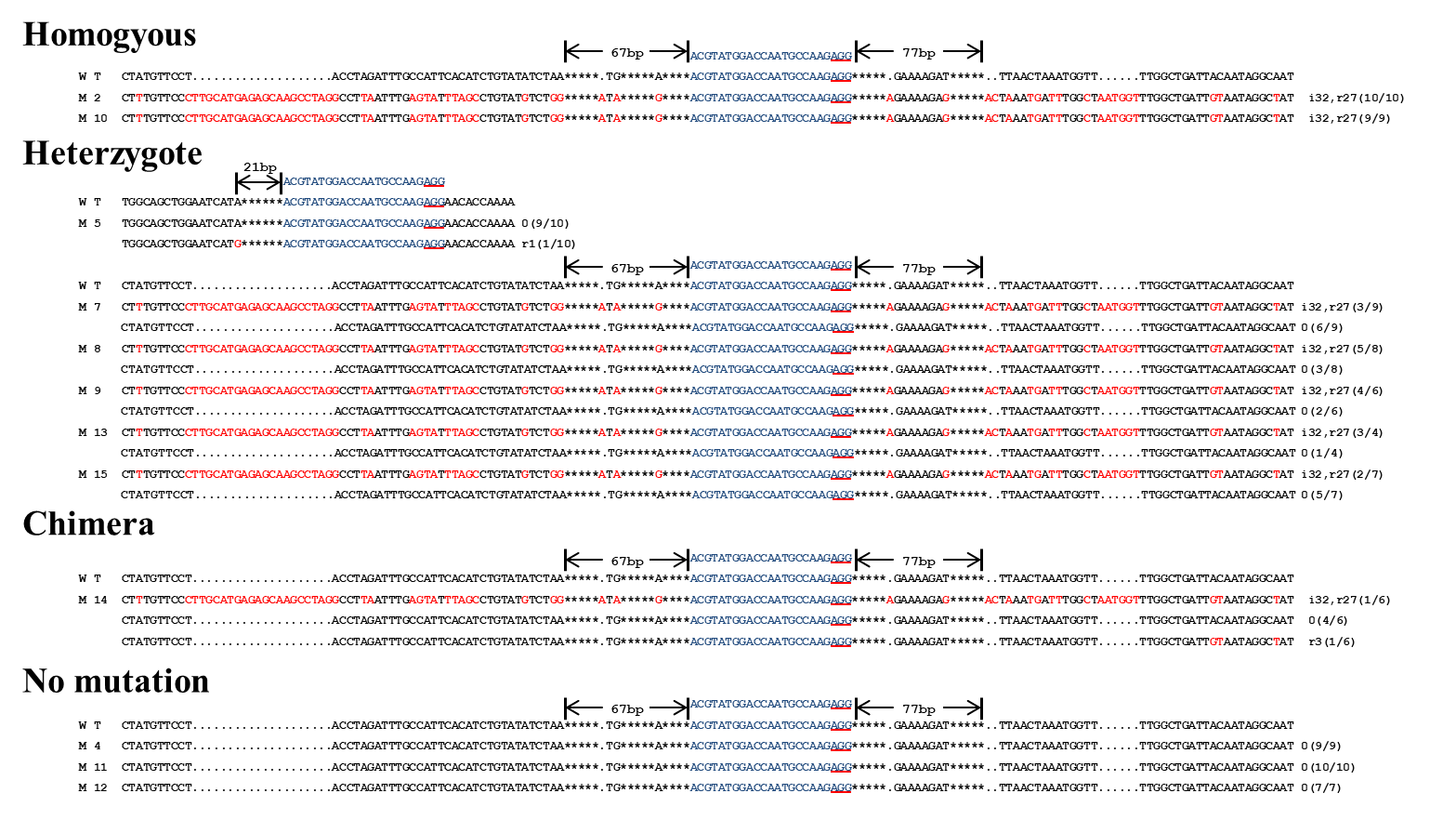
**

**Supplementary Fig. S3** CRISPR/Cas9-induced mutation detection in partial mutants not shown in Fig. 1b. The target sequence is indicated in blue, the PAM sequence (NGG) is underlined in red, mutated bases are indicated in a red font, and the asterisks indicate the spacing between bases. WT: wild-type plant; M #, # number of mutants; i #, # number of base insertions; r #, # number of base replacements.


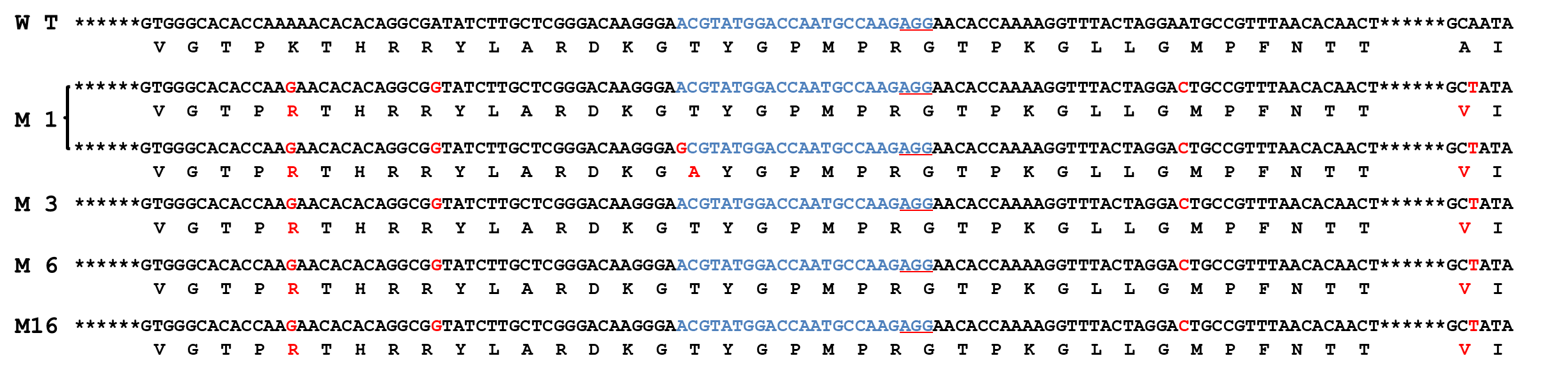


**Supplementary Fig. S4** Prediction of amino acid changes in Chinese kale *crtiso* mutants mediated by the CRISPR/Cas9 system. The target sequence is indicated in blue, the PAM sequence (NGG) is underlined in red, mutated bases and changed amino acids are indicated in a red font, and the asterisks indicate the intron. WT: wild-type plant; M #, # number of mutants.
